# Functional Characterization of a Flavonoid Glycosyltransferase in Sweet Orange (*Citrus sinensis*)

**DOI:** 10.3389/fpls.2018.00166

**Published:** 2018-02-15

**Authors:** Xiaogang Liu, Cailing Lin, Xiaodi Ma, Yan Tan, Jiuzhao Wang, Ming Zeng

**Affiliations:** ^1^College of Horticulture and Landscape Architecture, Southwest University, Chongqing, China; ^2^Key Laboratory of Horticulture Science for Southern Mountainous Regions, Ministry of Education, Chongqing, China

**Keywords:** sweet orange (*C. sinensis*), UGTs, flavonoid glycosylation, flavonoid rhamnosylation, flavonoid 7-*O*-glucosyltransferase, flavonoid 7-*O*-rhamnosyltransferase

## Abstract

Fruits of sweet orange (*Citrus sinensis*), a popular commercial *Citrus* species, contain high concentrations of flavonoids beneficial to human health. These fruits predominantly accumulate *O*-glycosylated flavonoids, in which the disaccharides [neohesperidose (rhamnosyl-α-1,2-glucose) or rutinose (rhamnosyl-α-1,6-glucose)] are linked to the flavonoid aglycones through the 3- or 7-hydroxyl sites. The biotransformation of the flavonoid aglycones into *O*-rutinosides or *O*-neohesperidosides in the *Citrus* plants usually consists of two glycosylation reactions involving a series of uridine diphosphate-sugar dependent glycosyltransferases (UGTs). Although several genes encoding flavonoid UGTs have been functionally characterized in the *Citrus* plants, full elucidation of the flavonoid glycosylation process remains elusive. Based on the available genomic and transcriptome data, we isolated a *UGT* with a high expression level in the sweet orange fruits that possibly encodes a flavonoid glucosyltransferase and/or rhamnosyltransferase. Biochemical analyses revealed that a broad range of flavonoid substrates could be glucosylated at their 3- and/or 7-hydrogen sites by the recombinant enzyme, including hesperetin, naringenin, diosmetin, quercetin, and kaempferol. Furthermore, overexpression of the gene could significantly increase the accumulations of quercetin 7-*O*-rhamnoside, quercetin 7-*O*-glucoside, and kaempferol 7-*O*-glucoside, implying that the enzyme has flavonoid 7-*O*-glucosyltransferase and 7-*O*-rhamnosyltransferase activities *in vivo*.

## Introduction

Flavonoids, such as flavanones, flavones, and flavonols, are among the most widespread groups of plant secondary metabolites and show a broad diversity of biological functions (Wang et al., 2007, 2011). Flavonoids are supposed to be beneficial to human health because of their anti-oxidant, anti-allergenic, and anti-inflammatory properties (Pietta, 2000; Ross et al., 2001). In plants, many flavonoid aglycones are glycosylated with pentoses and hexoses, resulting in the functional and structural diversity of flavonoids (Vogt and Jones, 2000; Jones and Vogt, 2001). The glycan moieties present in flavonoid glycosides may play key roles in increasing the aqueous solubility of these glycosides, thus improving their bioavailability in humans (Hollman et al., 1995; Hollman and Katan, 1999). Most flavonoid glycosylation reactions depend on UDP-sugar dependent glycosyltransferases (UGTs), which use UDP sugars as sugar donors (Dixon and Steele, 1999; Bowles, 2002; Bowles et al., 2005; Lairson et al., 2008) and contain a 44-amino-acid conserved sequence (the “PSPG motif”) in their C-terminal regions that is responsible for combining the UDP sugars (Gachon et al., 2005).

Several members of the UGT superfamily exhibit strict regiospecificity when performing glycosylation reactions on flavonoid substrates (Kramer et al., 2003; Cartwright et al., 2008); WsGT from *Withania somnifera*, for instance, was shown to glycosylate the 7-hydrogen sites of naringenin, apigenin, and luteolin (Kumar et al., 2013). In contrast, some UGTs can glycosylate substrates at multiple hydroxyl positions, such as the flavonoid glucosyltransferase from *Dianthus caryophyllus* that uses naringenin as a substrate to produce naringenin-4′-*O*-glucoside and naringenin-7-*O*-glucoside (Werner and Morgan, 2010). Another study has demonstrated the role of AtUGT74F1 in glycosylating quercetin at its 4′-, and 7-hydrogen sites (Lim et al., 2004). At present, *UGT*s have been cloned and functionally characterized in many plant species, which, based on their substrate preferences, can be largely classified into two major types. The first type directly adds a sugar to the aglycones of flavonoids and their derivatives; these include *Petunia hybrida* PGT8 (flavonoid 3-*O*-glucosyltransferase) and PH1 (flavonoid 5-*O*-glucosyltransferase) (Yamazaki et al., 2002), *Vitis vinifera* UFGT (flavonoid 3-*O*-glucosyltransferase) (Ford et al., 1998), *Rosa hybrida* RhGT1 (anthocyanin 3, 5-*O*-glucosyltransferase) (Ogata et al., 2005), and *Arabidopsis thaliana* UGT73C6 (flavonol 3-*O*-rhamnoside-7-*O*-glucosyltransferase) (Jones et al., 2003). The second type attaches a sugar to the glycogen of the flavonoid monoglycosides to produce diglycoside; this group includes *Ipomoea nil* 3GGT (anthocyanidin 3-*O*-glucoside-2″-*O*-glucosyltransferase) (Morita et al., 2005) and *Petunia hybrida* 3RT (anthocyanidin 3-*O*-glucoside-6″-*O*-rhamnosyltransferase) (Kroon et al., 1994).

**FIGURE 1:**
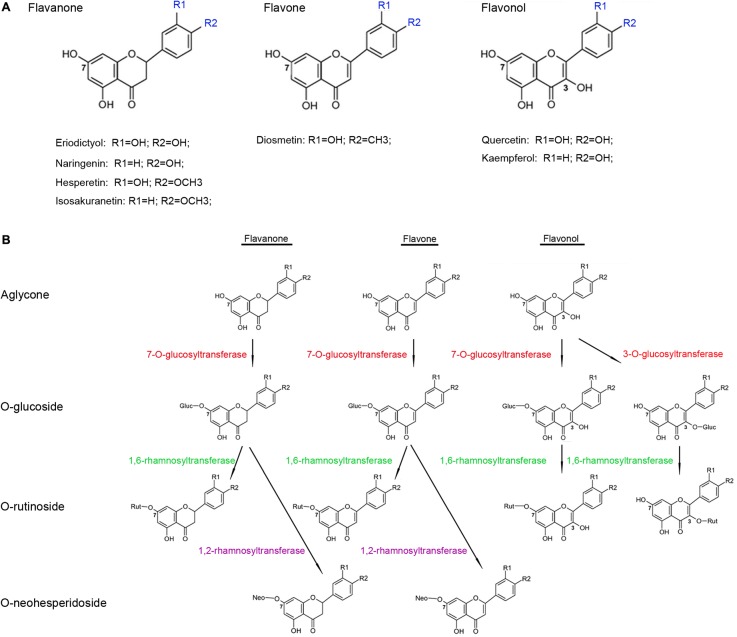
Structure **(A)** and glycosylation process **(B)** of flavonoid aglycones in *Citrus* plants.

*Citrus* fruits are known to accumulate high concentrations of flavonoid glycosides and have been widely used by the food-production sector as sources of these dietary chemicals. The biosynthetic pathway of the flavonoid glycosides is well-characterized in the *Citrus* plants, and most of the structural genes encoding the core enzymes have been identified from model plants (Tanaka et al., 2008). Most flavonoid glycosides in the *Citrus* plants are *O*-glycosides, in which the disaccharide is connected to the flavonoid aglycones by oxygen, and the most common disaccharides are neohesperidose (rhamnosyl-α-1,2-glucose) or rutinose (rhamnosyl-α-1,6-glucose). *Citrus* species, generally, have low levels of *C*-glycoside flavonoids, in which a C–C bond is directly formed between the anomeric carbon of the sugar moiety and the aromatic ring carbon of the flavonoid aglycones, in their fruits and leaves (Franz and Grün, 1983; Hultin, 2005). Biotransformation of the flavonoid aglycones into *O*-rutinosides or *O*-neohesperidosides in the *Citrus* plants typically consists of two glycosylation reactions involving a series of UGTs (Vogt and Jones, 2000; Li et al., 2001; Cantarel et al., 2009) (**Figure 1**). The first reaction is glucosylation at the 3- or 7-hydrogen sites of the flavonoid aglycones catalyzed by a 3-*O*- or a 7-*O*-glucosyltransferase, respectively (McIntosh et al., 1990; Berhow and Smolensky, 1995), and the subsequent reaction is catalyzed by rhamnosyltransferases, such as 1,6-rhamnosyltransferase catalyzing flavonoid 3-*O*- or 7-*O*-glucosides to produce flavonoid 3-*O*- or 7-*O*-rutinosides, respectively (Bar-Peled et al., 1993; Frydman et al., 2013), whereas, 1,2-rhamnosyltransferase only metabolizes flavonoid 7-*O*-glucosides (and not flavonoid 3-*O*-glucosides) into flavonoid 7-*O*-neohesperidoses (Lewinsohn et al., 1989; Frydman et al., 2004). Although several *UGT*s have been functionally characterized in the *Citrus* plants, their number is still relatively low given the large abundance of the *UGT*s in the genomes of these plants; for example, genome-wide analyses have identified 120 *UGT*s in *A. thaliana*, 164 *UGT*s in *Medicago truncatula* (Caputi et al., 2012), and 137 *UGT*s in *Linum usitatissimum* (Barvkar et al., 2012). Thus, further identification and characterization of these *UGT*s are important steps in understanding their roles in flavonoid glycosylation, as well as in the regulation of flavonoid accumulation in the *Citrus* fruits.

Here, we identified a new *UGT* (“*CsUGT76F1*”) isolated from the sweet orange fruits. Phylogenetic analysis suggested that CsUGT76F1 might function as a flavonoid glucosyltransferase and/or rhamnosyltransferase. Heterologous expression in *E. coli* was employed to investigate whether the recombinant protein functions as a flavonoid UGT, and to determine its substrate specificity and kinetic parameters toward various *Citrus* flavonoids. In addition, *CsUGT76F1* was overexpressed in tobacco to test its *in vivo* function.

## Materials and Methods

### Plant Materials and Growth Conditions

Sweet orange trees (*Citrus sinensis* ‘Valencia’) were grown in the greenhouse at the National Citrus Germplasm Repository, the Citrus Research Institute (CRI) of the Chinese Academy of Agricultural Sciences (CAAS), Chongqing, China. A total of seven developmental stages were collected from the fruit-setting period, which consisted of 10 DAB (days after full blooming), 30, 60, 90, 120, 150, and 180 DAB. All fruit samples were separated into two parts: peel (also called exocarp) and pulp (called endocarp). All samples were immediately frozen in liquid nitrogen and stored at -80°C. Tobacco plants (*Nicotiana tabacum*) used in the transgenic experiments were grown in the growth chamber at a constant temperature (28 ± 3°C), and were exposed to a 12/12 h cycle (light/dark).

### Sample Preparation and UPLC-Q-TOF-MS Conditions

Flavonoids in sweet orange were extracted using the procedure described by Yang et al. (2016). After drying at 40°C for 48 h, the peels and pulp were powdered and filtered through a 60-mesh screen. About 0.5 g of the powdered sample was ultrasonically extracted with 7 mL methanol at 200 W for 30 min. After centrifugation at 5,000 rpm for 10 min, the resulting supernatants were collected in a 15-mL tube. The process was repeated three times. The final volume was adjusted to make a consistent volume of 25 mL of methanol. About 0.4 mL of the extract solution was diluted with 0.6 mL deionized water and filtered through a membrane with 0.2-μm pore diameter and temporarily stored at 4°C in the refrigerator.

The tobacco flavonoid compounds were extracted by the following method: about 0.2 g of the powdered samples were extracted in solution (80% methanol, 19% water, 1% hydrochloric acid) by vortexing for 20 s followed by water-bath sonication for 30 min. After centrifugation at 6,000 rpm for 10 min, the resulting supernatants were collected and placed in a fresh 5-mL tube, then extracted twice with chloroform to remove chlorophyll. The extraction process was replicated three times, and the final volume was adjusted to 5 mL. Finally, the supernatants were filtered through a membrane with 0.2-μm pore diameter and temporarily placed at 4°C in the refrigerator.

Ultraperformance liquid chromatography analyses were implemented as described by Yang et al. (2016). After separation on an ACQUITY UPLC BEH C18 column (2.1 mm × 100 mm, 1.7 mm, United Kingdom), samples were scanned by a photodiode array detector with the absorption spectrum set from 240 to 400 nm. Xevo G2-S Q-TOF (Waters MS Technologies, Manchester, United Kingdom), a quadrupole, orthogonal acceleration, time-of-flight tandem mass spectrometer was used with an electron spray ionization source. Both positive and negative ion modes were employed to ionize the chemical compounds. The detection conditions were as follows: capillary voltage at 0.45 kV, cone voltage 40 V, source temperature 100°C, desolvation temperature 400°C, cone gas flow 50 L h^-1^, desolvation gas flow 600 L h^-1^, low energy 6 V, and high energy ramp 20–40 V. TOF-MS was set from 100 to 1000 m/z. The scan time was 0.2 s. Data was obtained using real-time collection (scan time 0.5 s, interval 15 s). Sample UPLC-Q-TOF-MS data were collected and processed using the Waters UNIFI 1.7 software.

### Cloning and qRT-PCR Analyses

Total RNA was extracted with the TaKaRa MiniBEST Universal RNA Extraction Kit (Takara Bio, China). cDNA synthesis was performed using the PrimeScript RT Master Mix Perfect Real-Time Kit (Takara Bio, China). According to the genomic data from Phytozome v12.1, primers were designed to obtain the full-length cDNA and to detect the relative expression levels. All PCR primer sequences are shown in **Supplementary Table S1**. The relative expressions of the genes were determined according to the 2^-ΔΔ*Ct*^ method (Livak and Schmittgen, 2001). Based on the analysis by geNorm (Vandesompele et al., 2002), three reference genes, citrus β*-actin*, *SDH1-1*, and *GAPDH*, were used to normalize the expressions of the candidate genes. Three replicates of the experiments were performed for each gene.

### Heterologous Expression in *E. coli*

The coding sequence of *CsUGT76F1* was amplified by PCR with forward and reverse primers (**Supplementary Table S1**), following which the PCR product was sub-cloned into the pMAL-c2X expression vector with a maltose-tag (New England Biolabs, Ipswich, MA, United States). The recombinant plasmid was introduced into *E. coli* NovaBlue (DE3) competent cells (Novagen, Schwalbach am Taunus, Germany). The positive clones were identified in 5 mL of lysogeny broth with 80 mg/L ampicillin for 8–12 h at 37°C. Two milliliters of *E. coli* culture were transferred to 300 mL of lysogeny broth containing 80 mg/L ampicillin and shaken at 200–250 rpm until an optical density (O.D.) of 0.6 at a wavelength of A_600_ was reached. Isopropyl-β-D-thiogalactopyranoside (IPTG) was employed to induce the expression of *CsUGT76F1*. After induction at 28°C for 48 h, the cells were precipitated by centrifugation and then disrupted by sonication for 20–25 min. The recombinant protein was purified via an amylose resin affinity chromatography system (New England Biolabs, E8201S).

Uridine diphosphate-rhamnose was synthesized using the procedure described by Shibuya et al. (2010) and Hsu et al. (2017). Full-length cDNA of the *RHM2*/*MUM4* gene (*At1g53500*) from *A. thaliana* was cloned into the expression vector pET21a and then introduced into the BL21-CodonPlus (DE3)-RIPL. The recombinant protein was prepared according to the method reported by Shibuya et al. (2010). The 80-μL of cell extract prepared from the *RHM2/MUM4*-expressing cells was incubated with 5 mM UDP-glucose, and 10 mM NADPH at 37°C for 2 h. The presence of UDP-rhamnose in the reaction mixture was identified via comparisons with the data in available literature (Hsu et al., 2017), and the concentrations were calculated using UDP-glucose as a reference.

### Enzymatic Assay and Optimization of Reaction Conditions

The standard reaction mixture for enzyme assay consisted 100 mM Tris-HCl (pH 7.5), 0.1% (v/v) β-mercaptoethanol, 2.5 mM sugar donors, 0.2 mM substrates, and 20 μg recombinant protein in a total volume of 50 μL. Firstly, flavonoid substrates were dissolved in dimethylsulfoxide (DMSO) to obtain a concentration of 15 mM. For substrate specificity analysis and to obtain optimal reaction conditions, flavonoid substrates dissolved in DMSO were then added to the reaction mixture with a final concentration of 0.2 mM. Although there was the possibility of slight precipitation of flavonoid aglycones occurring, such minor precipitation was believed to have no significant effect on the specificity analysis and the optimal reaction conditions that were derived from the presence or the maximum accumulation of the glycosylated flavonoids. A series of reaction mixtures containing different concentrations of the substrate (0.02, 0.04, 0.06, 0.08, 0.10, and 0.12 mM) were used to test the kinetic parameters of UGT. This selected range of substrate concentrations was far below the solubility of the flavonoid aglycones. The reaction was conducted at 30°C for 1 h and terminated by the addition of 60 μL pure methanol.

Optimal reactions were performed at varying pH and temperatures. Enzyme assays were conducted following the methods described above using different buffers. Three buffers were used to control the pH levels, including acid-sodium citrate buffer (pH 4.0–7.5), Tris-HCl buffer (pH 7.0–10.0), and NaHCO_3_–Na_2_CO_3_ buffer (pH 9.0–11.0). Reaction temperatures ranged from 10 to 70°C, with 5°C increments. Products from the enzymatic activity assay were analyzed using the UPLC-Q-TOF-MS system described above.

### Development of Binary Construct and Transformation of *CsUGT76F1* into Tobacco

The coding region of *CsUGT76F1* was amplified by PCR with forward and reverse primers (**Supplementary Table S1**). The PCR product was introduced into the vector pDONR207 using the Gateway BP Clonase Enzyme mix (Invitrogen, United States). Subsequently, *CsUGT76F1* was transferred into the expression vector pCB2004 using the Gateway LR Clonase system (Invitrogen, United States). The recombinant pCB2004-*CsUGT76F1* plasmid was transferred into the *Agrobacterium tumefaciens* EHA105-competent cells through electroporation. The positive cells were screened on the agar-solidified medium consisting 50 mg/L spectinomycin and 50 mg/L kanamycin, and genetic transformation was implemented via the leaf disk transformation method (Maiti et al., 1988). The transgenic plants were screened on Murashige and Skoog medium containing 25 mg/L phosphinothricin, and then identified using both genomic PCR and RT-PCR. Three lines with high transcript levels were used in the flavonoid profiling analyses.

## Results

### Identification of Flavonoids in Sweet Orange

**FIGURE 2:**
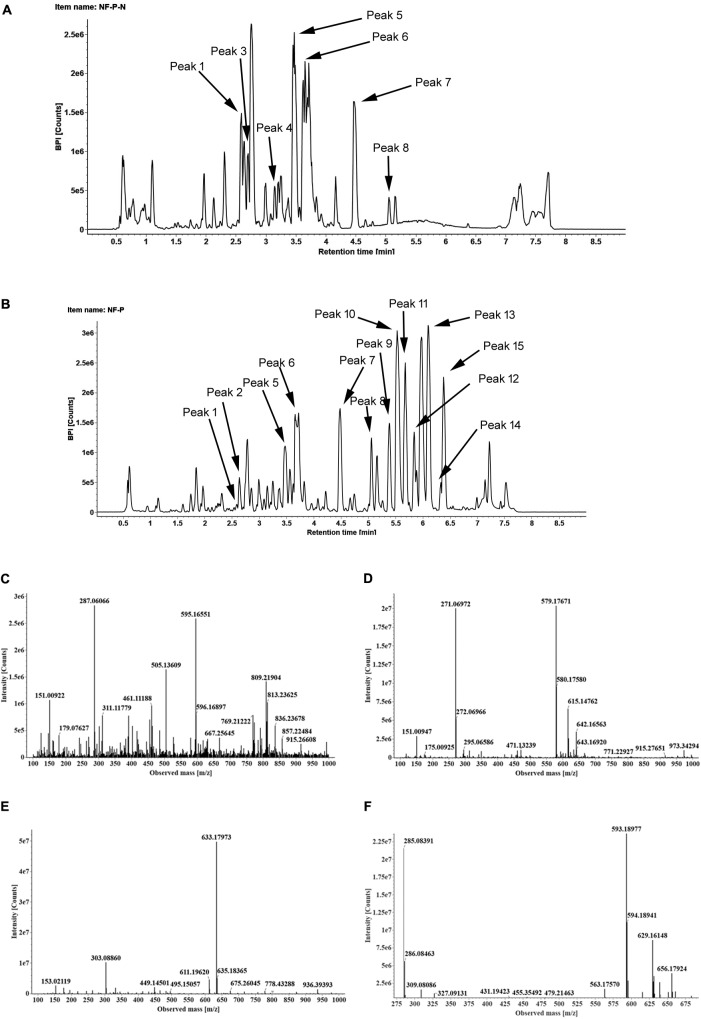
The BPI chromatograms corresponding to negative **(A)** and positive signals **(B)** of sweet orange peels at 180 DAB; The MS^2^ spectra of four *O*-glycosylated flavonoids, including eriocitrin **(C)**, narirutin **(D)**, hesperidin **(E)**, didymin **(F)**.

To identify the flavonoids and their derivatives, we employed UPLC-Q-TOF-MS in both positive and negative ion modes to analyze the peel and pulp extracts. The representative chromatograms of BPI corresponding to the negative and positive signals of sweet orange peels at 180 DAB are shown in **Figure 2**. A total of 13 flavonoids and their derivatives were detected and unambiguously characterized via comparisons with the reference standards and literature data (Abad-García et al., 2012; Yang et al., 2016) (MS data presented in **Supplementary Table S2**). Four flavonoid-*O*-glycosides in the fruit of sweet orange were identified, consisting eriocitrin (also called eriodictyol-7-*O*-rutinoside, peak 4), narirutin (naringenin-7-*O*-rutinoside, peak 5), hesperidin (hesperetin 7-*O*-rutinoside, peak 6), and didymin (isosakuranetin-7-*O*-rutinoside, peak 7). These flavonoid-*O*-glycosides often contain a disaccharide (rhamnosyl-α-1,6-glucose) linked to an aglycone through the *C*-7 hydroxyl group. In addition, two flavonoid-*C*-glycosides- apigenin-6,8-di-*C*-glucoside (peak 1) and chrysoeriol-6,8-*C*-glucoside (peak 3), were also identified. The polymethoxylated flavones are found almost exclusively in the *Citrus* species. Our results revealed the presence of seven polymethoxylated flavones in the fruit of sweet orange, consisting of isosinensetin (peak 9), sinensetin (peak 10), dihydroxy-tetramethoxyflavone nobiletin (peak 11), 5, 7, 8, 4′-tetramethoxyflavone (peak 12), 3, 5, 6, 7, 8, 3′, 4′-heptamethoxyflavone (peak 13), 5-hydroxy-6, 7, 8, 3′, 4′-pentamethoxyflavone (peak 14), and tangeretin (peak 15). Overall, these four flavonoid *O*-glycosides described above, viz., eriocitrin, narirutin, hesperidin, and didymin, appear to be ubiquitously present, and consequently detected, in all sweet orange fruit peels and pulp (with the exception of didymin in pulp).

### cDNA Cloning, Sequence Comparison, and Phylogenetic Analysis

One hundred and seven *Arabidopsis* UGTs were employed as a query for BLASTP searches against the *C. sinensis* genome^1^, which identified 123 putative UGTs sorted by *e* value (1*e*^-5^). Based on previous transcriptome data sets (Wu et al., 2014; Huang et al., 2016; Wang J. et al., 2016), 10 putative *UGT* genes were found to be highly expressed in sweet orange.

**FIGURE 3:**
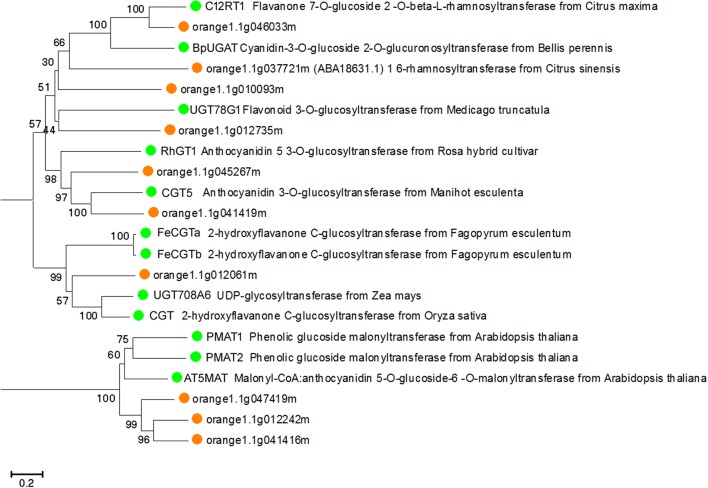
Phylogenetic tree based on the full-length proteins of 10 *Citrus* and 12 functionally characterized UGT proteins. Gene names and accession numbers: C12RT1 (AY048882), BpUGAT (AB190262), UGT78G1 (XM_003610115), Cs1,6Rhat (DQ119035), RhGT1 (AB201048), CGT5 (X77462), FeCGTa (AB909375), FeCGTb (AB909376), UGT708A6 (NM_001139178), CGT (FM179712), PMAT1 (NM_123267), PMAT2 (NM_113889), AT5MAT (NM_113880). Bootstrap values are shown above the nodes.

To predict whether the putative *Citrus* UGTs are involved in flavonoid glycosylation, an unrooted NJ phylogenetic tree (**Figure 3**) was constructed based on the full-length proteins of 10 *UGT*s and 12 functionally characterized flavonoid UGTs. From the phylogenetic trees, three putative UGTs (orange1.1g046033m, orange1.1g010093m, and orange1.1g012735m) were clustered with C12RT1, BpUGAT, UGT78G1, and Cs1,6Rhat (encoded by *orange1.1g037721m*). These UGTs are known to function as glucosyltransferase, glucuronosyltransferase, or rhamnosyltransferase of flavonoids. Alignment analysis revealed the presence of a conserved PSPG box in the *C*-terminus of all UGTs in this cluster (**Supplementary Figure S1**).

**FIGURE 4:**
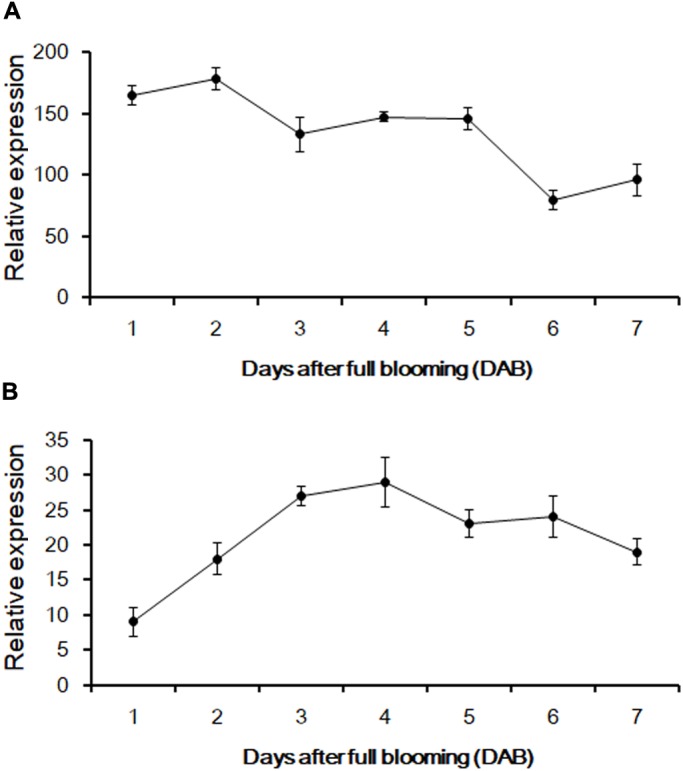
Relative expression of *CsUGT76F1* in sweet orange peels **(A)** and pulp **(B)** during fruit development. Data represent means with standard deviations (*n* = 5).

According to the genomic data obtained from Phytozome v12.1, PCR amplification was performed to produce the full-length cDNA of three genes from the fruit of sweet oranges. Only *orange1.1g012735m* could be obtained, however; this gene is 1,495 bp long and contains a 3′ untranslated region (UTR) of 41 bp, a 5′ UTR sequence of 80 bp, and an ORF of 1,374 bp that encodes a 475-amino acid protein. The base sequence information of *orange1.1g012735m* could be retrieved from Phytozome v12.1 and the gene, *CsUGT76F1*, was named according to the UGT committee nomenclature protocols. qRT-PCR was employed to investigate the expression abundance of *CsUGT76F1* in fruit pulp and peels during fruit development (**Figure 4**). Transcripts of *CsUGT76F1* had higher levels of expression in peels than in pulp. In peels, *CsUGT76F1* was downregulated during fruit development and ripening, whereas in pulp, the transcript was upregulated initially and then gradually decreased with fruit ripening.

### Enzymatic Assays and Product Identification

**FIGURE 5:**
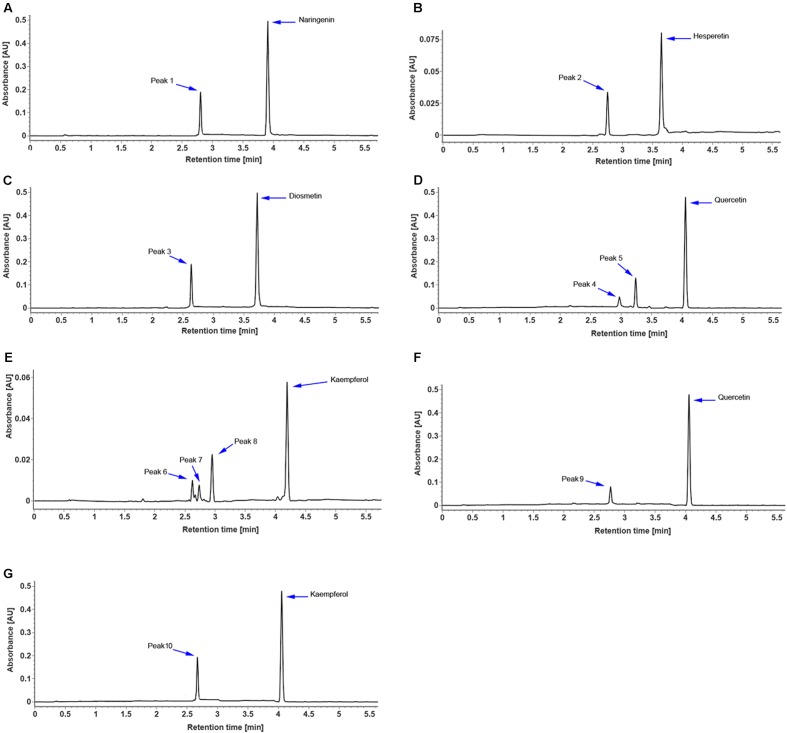
UPLC chromatogram of reaction products of recombinant CsUGT76F1, using UDP-glucose as the sugar donor, toward naringenin **(A)**, hesperetin **(B)**, diosmetin **(C)**, quercetin **(D)**, and kaempferol **(E)**, and using UDP-rhamnose as the sugar donor, toward quercetin **(F)**, and kaempferol **(G)**. Peaks 1–10 indicate enzymatic products listed in **Supplementary Table S3**.

The full-length coding region of *CsUGT76F1* was transferred into a pMAL-C2X expression vector with a maltose-binding protein (MBP) tag and introduced into *E. coli.* The recombinant CsUGT76F1 was purified by a MBP tag purification system. Initial *in vitro* assays using UDP-glucose as the sugar donor indicated that the protein could catalyze the glucosyl transfer to the 7-hydrogen sites of hesperetin, naringenin, and diosmetin (**Figures 5A–C**). The biotransformation products were verified using UPLC-Q-TOF-MS by comparing them with reference standards, the mass of the molecular ion, and the resulting fragments (**Supplementary Table S3**). Subsequently, substrate specificity studies for the recombinant protein with flavonols were also conducted, including quercetin and kaempferol, the glycosylated products of which had been previously identified in other *Citrus* plants (Abad-García et al., 2012; Wang S. et al., 2016). The results demonstrated that the recombinant UGT was able to glucosylate quercetin at the 3- or 7-hydrogen position (**Figure 5D**), but using kaempferol as substrate, three products— kaempferol 3,7-*O*-diglucoside, kaempferol 3-*O*-glucoside, and kaempferol 7-*O*-glucoside, were detected (**Figure 5E**).

In addition, the sugar-donor specificities for the recombinant protein were also investigated, including UDP-rhamnose, UDP-galactose, and UDP-xylose. The results demonstrated that the recombinant UGT can only accept UDP-rhamnose to rhamnosylate the 7-hydrogen position of quercetin and kaempferol (**Figures 5F,G**), but not to catalyze the rhamnosylation of hesperetin, naringenin, and diosmetin. Since flavonoid-*O*-glycosides in the *Citrus* plants often contain a disaccharide (rhamnosyl-α-1,6-glucose or rhamnosyl-α-1,2-glucose), which is sequentially added to the flavonoid aglycones, we further tested whether the recombinant UGT was able to rhamnosylate flavonoid-*O*-monoglycosides, including hesperetin-7-*O*-glucoside, naringenin-7-*O*-glucoside, diosmetin-7-*O*-glucoside, quercetin-3 and 7-*O*-glucoside, and kaempferol-3 and 7-*O*-glucoside. No flavonoid *O*-rutinoside (rhamnosyl-α-1,6-glucose) or *O*-neohesperidose (rhamnosyl-α-1,2-glucose) products were detected. These results indicated that CsUGT76F1 *in vitro* could function as flavonoid 3-*O*-, 7-*O*-, 3,7-*O*-glucosyltransferase, and 7-*O*-rhamnosyltransferase.

**FIGURE 6:**
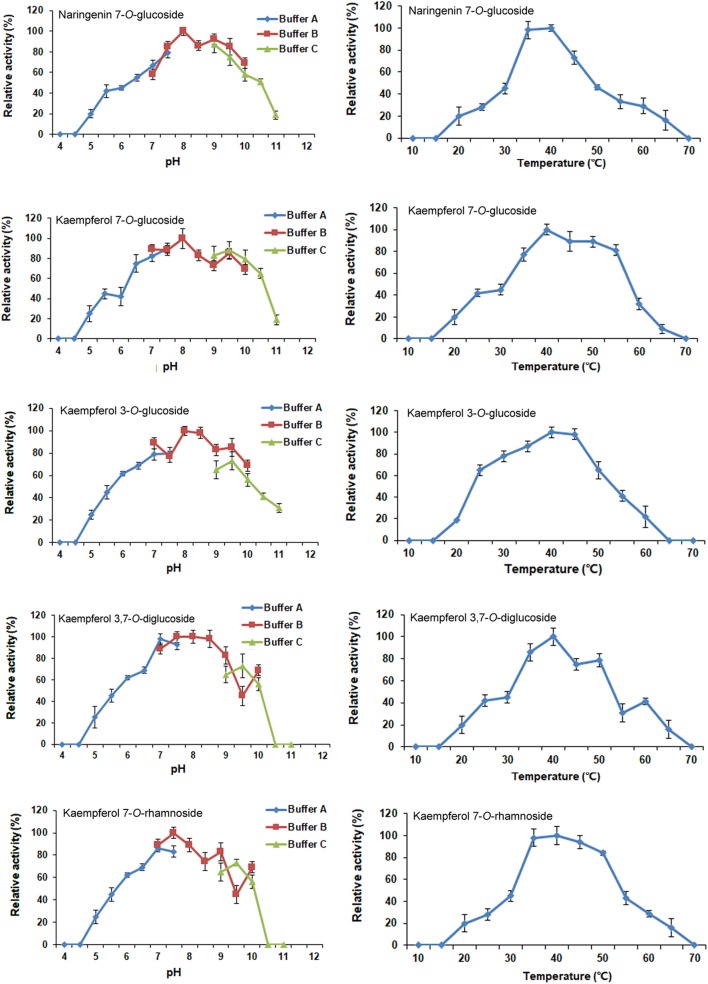
Optimization of reaction pH and temperature of recombinant CsUGT76F1. Buffer A: acid-sodium citrate buffer (pH 4.0–7.5). Buffer B: Tris-HCl buffer (pH 7.0–10.0). Buffer C: NaHCO_3_–Na_2_CO_3_ buffer (pH 9.0–11.0).

The optimal reaction conditions for CsUGT76F1 were determined using naringenin and kaempferol as substrates, and UDP-glucose and UDP-rhamnose as the sugar donors (**Figure 6**). The pH and temperature of the reaction buffer ranged from 4.0–11.0 to 10–70°C, respectively. The maximum levels of naringenin 7-*O*-glucoside product accumulation occurred at pH 8.0 and a temperature of 40°C; for kaempferol 7-*O*- and 3-*O*-glucoside, the maximum accumulations occurred at pH 8.0 and 40–45°C temperature, and pH 8.0–8.5 and 40°C temperature, respectively; and for kaempferol 3,7-*O*-diglucoside, the maximum accumulation occurred at pH 7.5–8.5 and temperatures of 35–45°C. The maximum accumulation of the kaempferol 7-*O*-rhamnoside product could be observed at pH 7.5 and a temperature of 35–40°C.

The kinetic parameters of CsUGT76F1 were further investigated on the flavonoid substrates at pH 8.0 and 40°C (**Table 1**). The enzyme showed the highest affinity toward the substrates hesperetin (*K_m_* = 15.16 μM) and naringenin (*K_m_* = 20.41 μM) compared to that toward diosmetin (*K_m_* = 43.27 μM), quercetin (*K_m_* = 36.78 μM), and kaempferol (*K_m_* = 28.09 μM). However, the enzyme used diosmetin (*k_cat_* = 1.60 s^-1^) most efficiently, followed by naringenin (*k_cat_* = 0.71 s^-1^) and hesperetin (*k_cat_* = 0.77 s^-1^). The *kcat* values for the quercetin and kaempferol were 0.58 and 0.46 s^-1^, respectively. The *k*_cat_/*K*_m_ ratio was the highest for hesperetin (*kcat*/*Km* = 50.39 M^-1^ s^-1^), followed by that for naringenin (*kcat*/*Km* = 34.79 M^-1^ s^-1^), diosmetin (*kcat*/*Km* = 36.98 M^-1^ s^-1^), quercetin (*kcat*/*Km* = 15.77 M^-1^ s^-1^), and kaempferol (*kcat*/*Km* = 16.38 M^-1^ s^-1^).

**Table 1:** Kinetic parameters for the recombinant CsUGT76F1.

Substrate	*K_m_* (*μ*M)	*k_cat_* (s^-1^)	*kcat*/*Km* (M^-1^ s^-1^)
Hesperetin	15.16 ± 6.280	0.77 ± 0.079	50.39 × 10^3^± 13.12 × 10^3^
Naringenin	20.41 ± 3.568	0.71 ± 0.033	34.79 × 10^3^± 4.72 × 10^3^
Diosmetin	43.27 ± 2.114	1.60 ± 0.068	36.98 × 10^3^± 2.77 × 10^3^
Quercetin	36.78 ± 4.316	0.58 ± 0.071	15.77 × 10^3^± 8.90 × 10^3^
Kaempferol	28.09 ± 3.742	0.46 ± 0.029	16.38 × 10^3^± 12.36 × 10^3^


*Data are the means ± SD of three independent experiments.*

### Heterologous Expression in Tobacco

**FIGURE 7:**
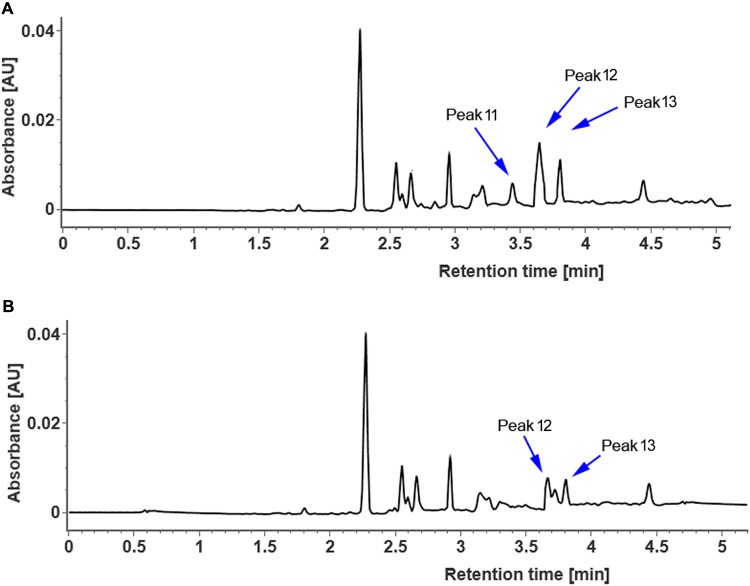
UPLC chromatogram of flavonoid glycosides extracted from transgenic **(A)** and control **(B)** tobacco. Peaks 11–13 indicate enzymatic products listed in **Supplementary Table S3**.

In order to clarify whether CsUGT76F1 functions as a rhamnosyltransferase, the coding region of *CsUGT76F1* under the 35S promoter was introduced into tobacco plants, with the positive transgenic plants identified using antibiotic selection and flavonoid compounds extracted from the leaves of transgenic and control tobacco. Compared with control tobacco, one new flavonol glycoside (**Figure 7**, peak 11) was produced in transgenic tobacco, and two other flavonol glucosides were significantly higher (peak 12 and peak 13). Based on the results of both the UPLC-Q-TOF-MS analysis and previous studies, the peak 11 product was identified as quercetin 7-*O*-rhamnoside, peak 12 was quercetin 7-*O*-glucoside, and peak 13 was kaempferol 7-*O*-glucoside (**Supplementary Table S3**). Higher accumulation of quercetin 7-*O*-rhamnoside, quercetin 7-*O*-glucoside and kaempferol 7-*O*-glucoside in the transgenic plants demonstrated that CsUGT76F1 had flavonoid 7-*O*-glucosyltransferase and 7-*O*-rhamnosyltransferase activities *in vivo*.

## Discussion

Flavonoid *O*-neohesperidosides, such as poncirin, naringin, neoeriocitrin, and neohesperidin, were previously found in peels and juices of sweet orange (Gattuso et al., 2007; Wang et al., 2008; Khan et al., 2010). High concentrations of naringin were found in the sweet orange fruits (Destani et al., 2013; Luengo et al., 2013). Moreover, extremely high concentrations of the flavonoid neohesperidin were detected in the peel and juice of Liucheng [*C. sinensis* (L.) Osbeck] and Murcott (*C. reticulata* × *C. sinensis*) (Wang et al., 2007). However, we detected only the four flavonoid 7-*O*-rutinosides (eriocitrin, narirutin, hesperidin, and didymin) in the fruit of sweet orange (**Figure 2**), whereas, the flavonoid 7-*O*-neohesperidosides were all found at concentrations below the detectable levels by UPLC-Q-TOF-MS. These four flavonoid *O*-rutinosides were found to exist in the sweet orange fruit peels and pulp (with the exception of didymin in pulp).

In plants, UGTs appear to be the central players in determining the chemical diversity of flavonoids. Thus, understanding the biochemical features of UGT enzymes is crucial for both crop genetic improvement and the production of recombinant enzymes for the biosynthesis of desired flavonoids that have medicinal and nutritional application. The biochemical properties of several UGTs have been characterized from *Citrus* species over the past several years. For example, in transgenic tobacco, by feeding with flavonoid aglycones, C12RT1 from pummelo and Cs1,6Rhat from sweet orange showed functions as rhamnosyltransferases that add rhamnose to flavanone-7-*O*-glucosides (Bar-Peled et al., 1993; Frydman et al., 2004). Ohashi et al. (2016) further conducted direct enzyme kinetic studies and determined substrate preference *in vitro*. The results demonstrated that Cs1,6Rhat appeared to be somewhat promiscuous with respect to substrate preference. But the number of functionally characterized *UGT*s is still relatively low given the large abundance of *UGT*s in the genomes of *Citrus* plants.

Phylogenetic analysis revealed CsUGT76F1 to be most closely clustered with C12RT1, BpUGAT, UGT78G1, and Cs1,6Rhat (encoded by *orange1.1g037721m*) (**Figure 3**), but these enzymes differ considerably in their substrate specificities. Of the enzymes in this cluster, C12RT1 and Cs1,6Rhat showed *in vitro* rhamnosyl transferring activities toward flavonoid-7-*O*-glucoside (Frydman et al., 2004, 2013; Ohashi et al., 2016), UGT78G1 catalyzes the glucosylation of flavonoids at the 3-*O*- hydroxyl site (Modolo et al., 2007, 2009), and BpUGAT may function as an anthocyanin glucuronosyltransferase to transfer glucuronate onto cyanidin 3-*O*-glucoside (Sawada et al., 2005; Osmani et al., 2008). Such incongruence between the phylogenetic position and substrate specificities has been found in other UGTs, including grape VLOGT2 and onion UGT73G1 and onion UGT73J1 (Kramer et al., 2003; Hall et al., 2011). These results support the proposition that the functions and specificities of UGTs is perhaps not accurately determined based on their protein sequences alone (Dhaubhadel et al., 2008). Thus, the coupling of phylogenetic analyses with experimental analyses is generally regarded as the most efficient approach for identifying UGT functions.

The recombinant CsUGT76F1 recognizes hesperetin, naringenin, diosmetin, quercetin, and kaempferol as substrates (**Table 1**). These results are is not completely consistent with those presented by the *in vivo* assays demonstrating that CsUGT76F1 prefers to catalyze the glucosylation of flavonols (**Figure 7**). This is because, the relative concentrations of the potential substrates *in vivo* might be one of the most critical factors that determine this enzyme’s activity. Another feature of the recombinant enzyme is its ability to catalyze the transfer of glucose onto the 3-hydrogen site of quercetin and kaempferol *in vitro*, but no significant increases in flavonoid-3-*O*-glycosides were detected in transgenic tobacco. In plants, glycosylation producing flavonoid compounds can occur on individual hydroxyl groups of the aglycon, or multiple hydroxyl groups simultaneously. Overall, only a few UGTs catalyzed multiple hydroxyl glycosylations. In *Arabidopsis*, 91 UGTs were isolated, 29 of which could glucosylate quercetin solely at individual hydroxyl sites, whereas, only one (AtUGT88A1) could glucosylate 3-, 7-, 3′-, and 4′-OH of quercetin simultaneously (Lim et al., 2004). Strawberry FaGT6 has been shown to catalyze quercetin to form 3-*O*-glucoside, as well as minor amounts of 7-*O*-, 4′-*O*-, 3′-*O*-monoglucoside and a diglucoside (Griesser et al., 2008); similarly, FaGT7 catalyzed quercetin to form quercetin 3-*O*-, 4′-*O*-, 7-*O*-, and 3′-*O*-monoglucoside, but not a diglucoside (Griesser et al., 2008). In addition to the characteristics described above, the pH optima of the recombinant enzyme seem unlikely to be of physiological importance *in vivo*. But similar results have been reported in rCsUGT75L12 and CsUGT73A20 from tea plants, and UGT78K1 from black soybean showed high pH optima in *in vitro* biochemical assays, but was demonstrated to play significant roles in the biosynthesis of flavonoids *in vivo* (Kovinich et al., 2010; Dai et al., 2017; Zhao et al., 2017). These UGTs show significant enzyme optima; as such, they are good candidates to engineer flavonoid diversity.

Previous works on flavonoid glycosylation in *Citrus* indicated that different UGTs are responsible for the sequential transfer of glucose and rhamnose in the formation of flavonoid diglycosides (Jourdan and Mansell, 1982; Kleinehollenhorst et al., 1982). Glucosyltransferases were capable of glucosylating flavonoids, resulting in the production of flavonoid-*O*-glucosides, which subsequently were converted to flavonoid-*O*-rutinosides by C12RT1 or Cs1,6Rhat (**Figure 1**). In this study, transgenic tobacco plants had higher concentrations of quercetin 7-*O*-glucoside, kaempferol 7-*O*-glucoside, and quercetin 7-*O*-rhamnoside (**Figure 7**), suggesting that CsUGT76F1 could function as a flavonoid 7-*O*-glucosyltransferase and 7-*O*-rhamnosyltransferase *in vivo*. These findings were also supported by the findings of the *in vitro* assays. But it is necessary to highlight that CsUGT76F1 only glycosylated flavonoid aglycones, unlike C12RT1 and Cs1,6Rhat that could recognize flavonoid monoglycosides as substrates.

## Conclusion

CsUGT76F1 can be identified as a flavonoid 7-*O*-UGT. Biochemical analysis in conjunction with *in vivo* data revealed its involvement in the biosynthesis of flavonoid 7-*O*-glucosides and 7-*O*-rhamnosides. Moreover, this enzyme exhibits broad substrate specificity toward flavonoids, including naringenin, hesperetin, diosmetin, kaempferol, and quercetin, present in *Citrus* species. Because of its broad substrate specificity and low regiospecificity, this recombinant enzyme promises to be an attractive choice for the engineering of flavonoid diversity.

## Author Contributions

MZ and XL designed the experiments; XL, CL, XM, YT, and JW performed the experiments and carried out the analysis; XL and CL wrote the manuscript.

## Conflict of Interest Statement

The authors declare that the research was conducted in the absence of any commercial or financial relationships that could be construed as a potential conflict of interest.

## Footnotes

1 https://phytozome.jgi.doe.gov/pz/portal.html, Phytozome 12

## Supplementary Material

**FIGURE S1 |** Amino acid sequence alignment and PSPG box of CsUGT76F1 and six homologies. The multiple alignment was performed using ClustalW2 software. PSPG box logo was produced by using MEME software (http://meme-suite.org) (Bailey et al., 2009).

**TABLE S1 |** Primers sequences used in experiments.

**TABLE S2 |** Identification of flavonoids in fruit peels of sweet orange by UPLC-Q-TOF-MS.

**TABLE S3 |** MS analyses of products in **Figures 6**, **7**.

## Abbreviations

BPI: basic peak ion
qRT-PCR: real-time quantitative reverse transcriptional-polymerase chain reaction
UGTs: uridine diphosphate (UDP)-sugar dependent glycosyltransferases
UPLC-Q-TOF-MS: ultraperformance liquid chromatography-quadrupole time-of-flight mass spectrometry
