# Supplementary figures and images for: Functional Characterization of a Flavonoid Glycosyltransferase in Sweet Orange (Citrus sinensis)
Source: Front Plant Sci. 2018 Feb 15;9:166. doi: 10.3389/fpls.2018.00166 (PMC5818429; doi:10.3389/fpls.2018.00166)

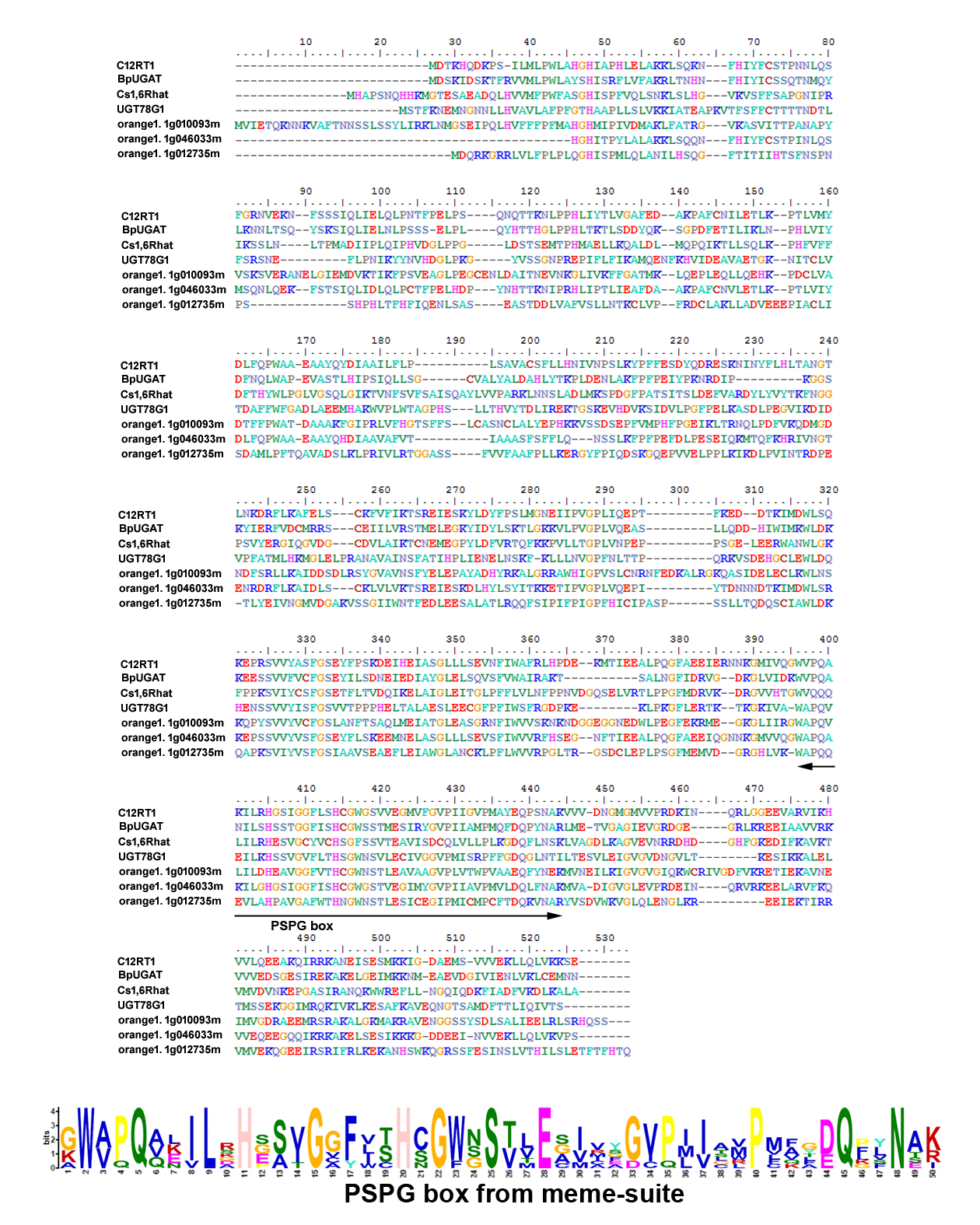

Supplement: Supplementary file 1 [file Image_1.TIF]
