# Supplementary material for: Functional Characterization of a Flavonoid Glycosyltransferase in Sweet Orange (Citrus sinensis)
Source: Front Plant Sci. 2018 Feb 15;9:166. doi: 10.3389/fpls.2018.00166 (PMC5818429; doi:10.3389/fpls.2018.00166)
Supplement: Supplementary file 2 [file Table_1.DOCX]

**Table S1. Primers sequences used in experiments.**

| **Purpose** | **Primer sequence (5'-3')** |
| --- | --- |
| **Cloning the full length of *orange1.1g012735m*** | Forward: GGGCCACAGCTAGAAAGACGCG |
|  | Reverse: ACCAAACTTGCGGGAAGAGTATTT G |
| **Real-time PCR of *orange1.1g012735m*** | Forward: GAACCTCTTGAGCAGCTCTTG |
|  | Reverse: CTCGTCAGCTTGATCTCACCAG |
| **Internal reference gene (*β*-actin;NCBI:** **BQ623464.1; Phyzome: *orange1.1g040984m*)** | Forward: CATCCCTCAGCACCTTCC |
|  | Reverse: CCAACCTTAGCACTTCTCC |
| **Internal reference gene (SDH1-1; NCBI: EY722231;**  **Phyzome: *orange1.1g046556m*)** | Forward: CAGGATGCTATTCAATATATGTGTAG |
|  | Reverse: CCA AAATCAAGACTTTGACCGCC |
| **Internal reference gene (GAPDH; NCBI:**  **XM_006476919.2; Phyzome: *orange1.1g024629m*)** | Forward: GTCTTGCCTGCTTTGAATGG |
|  | Reverse: GCATCCTTCTCCAGCCTCAC |
| **Protein biosynthesis for *RHM2/MUM4*** |  |
| Forward Primer with *Bam* H I site  Reverse Primer with *Xho* I site | Forward: AAGCGTGGATCCATGGATGATACTACGTATAAG  Reverse: TCATTCCTCGAGGGTTCTCTTGTTTGGTTC |
| **Protein biosynthesis for *orange1.1g012735m***  Forward Primer with *Bam* H I site  Reverse Primer with *Sal* I site | Forward: CGCGGATCCATGGTCATCGAAACACAAAAAAAT |
|  | Reverse: ACGCGTCGACTTAGCTGCTTTGATGGCGACTTA |
| **Heterologous expression in Tobacco** | Forward: GGGGACAAGTTTGTACAAAAAAGCAGGCTATGGTCATCGAAACACAAAAAAAT |
|  | Reverse: GGGGACCACTTTGTACAAGAAAGCTGGGTTTAGCTGCTTTGATGGCGACTTA |
