# Supplementary material for: Functional Characterization of a Flavonoid Glycosyltransferase in Sweet Orange (Citrus sinensis)
Source: Front Plant Sci. 2018 Feb 15;9:166. doi: 10.3389/fpls.2018.00166 (PMC5818429; doi:10.3389/fpls.2018.00166)
Supplement: Supplementary file 3 [file Table_2.DOCX]

**Table S2. Identification of flavonoids in fruit peels of sweet orange by UPLC-Q-TOF-MS.**

| **Peak No.** | **RT**  **(min)** | **Molecular**  **formula** | **MS data on [M+H]^+^**  **(m/z)** | **MS data on [M-H]^-^**  **(m/z)** | **MS^2^ focused on [M+H]^+^ (m/z)** | **MS^2^ focused on [M-H]^-^ (m/z)** | **Identification** |
| --- | --- | --- | --- | --- | --- | --- | --- |
| 1 | 2.58 | C_17_H_30_O_15_ | 595.16514  (0.61) | 593.15108  (0.92) | 617.1467[M+Na]^+^  577.1563[M+H-H_2_O]^+^  475.1122[M+H-C_4_H_8_O_4_- H_2_O]^+^  457.1122[M+H-C_4_H_8_O_4_-H_2_O]^+^ | 503.1222[M-H-C_3_H_6_O_3_]^-^  473.1018[M-H-C_4_H_8_O_4_] ^-^  383.0770[M-H-C_4_H_8_O_4_-C_3_H_6_O_3_] ^-^  353.0661[M-H-2C_4_H_8_O_4_] ^-^ | Apigenin-6,8-di-*C*- glucoside |
| 2 | 2.63 | C_33_H_42_O_19_ | nd | 741.21826  (0.62) | 765.2201[M+Na]^+^  435.1247[M+H-Glc-Rha]^+^ | 787.2281[M+HCOO]^-^  433.1138[M-H-Glc-Rha] ^-^  271.0599[M-H-2Glc-Rha] ^-^ | Narirutin-4’-  glucoside |
| 3 | 2.70 | C_28_H_32_O_16_ | 625.1763  (-0.6) | 623.15938  (-2.38) | 647.1618[M+Na]^+^  607.1651[M+H-H_2_O] ^+^  487.1218[M+H-C_4_H_8_O_4_-H_2_O^+^ | 593.1495[M-H-CH_2_O]^-^  533.1296[M-H-C_3_H_6_O3]^-^  503.1181[M-H-C_4_H_8_O_4_]^-^  485.1661[M-H-C_4_H_8_O_4_-H_2_O]^-^  413.1053[M-H-C_4_H_8_O_4_- C_3_H_6_O_3_]^-^  383.1491[M-H-2C_4_H_8_O_4_]^-^ | Chysoeriol-6,8-C- glucoside |
| 4 | 3.08 | C_27_H_32_O_15_ | nd | 595.16555  (-1.28) | nd | 475.1436[M-H-C_4_H_8_O_4_]^-^  287.0548[M-H-Glc-Rha]^-^ | Eriocitrin |
| 5 | 3.47 | C_27_H_32_O_14_ | 581.18568  (-0.08) | 579.17853  (6.60) | 603.1683[M+Na]^+^  419.1336[M+H–Glc]^+^  273.0759[M+H–Glc–Rha]^+^  147.0653,153.0184 | 625.17710[M+HCOO]^-^  313.0712[M-H-C_4_H_8_O_4_-Rha]^-^  271.0605[M-H-Glc-Rha]^-^  349.1912, 151.0036 | Narirutin |
| 6 | 3.65 | C_28_H_34_O_15_ | 611.19639  (-0.30) | 609.18253  (0.036) | 633.1785[M+Na]^+^  465.1383[M+H-Rha]^+^  449.1434[M+H -Glc]^+^  431.1334[M+H-Glc-H_2_O]^+^  413.1229[M+H-Glc-2H_2_O]^+^  303.0860[M+H-Glc-Rha]^+^  281.0655,195.0289, 153.0183 | 655.1876[M+HCOO]^-^  343.0792[M-H-Glc-C_4_H_8_O_4_]^-^  301.0717[M-H-Glc-Rha]^-^  286.0475[M-H-Glc-Rha-CH_3_]^-^  151.0036 | Hesperidin |
| 7 | 4.50 | C_28_H_34_O_14_ | 595.20115  (-0.68) | 593.18710  (-0.48) | 617.1840[M+Na]^+^  449.1469[M+H-Rha]^+^  433.1469[M+H-Glc]^+^  287.0914[M+H-Glc-Rha]^+^ | 639.1921[M+HCOO]^-^  285.0781[M-H- Glc-Rha]^-^ | Isosakuranetin-7-*O*-rutinoside |
| 8 | 5.06 | C_36_H_53_N_7_O_9_ | 728.39534  (-1.0) | 726.37767  (0.34) | 750.3814[M+Na]^+^  537.2082,226.9504,242.2851,  627.3355,393.2962,288.9230 | 772.3816 [M+HCOO]^-^  590.3301, 455.3552, 343.0861 | Citrusin III |
| 9 | 5.38 | C_20_H_20_O_7_ | 373.13022  (2.40) | nd | 395.1097[M+Na]^+^  358.1034[M+H-CH_3_]^+^  343.0811[M+H-2CH_3_]^+^  241.06987 | nd | Isosinensetin |
| 10 | 5.54 | C_20_H_20_O_7_ | 373.13315  (2.40) | nd | 395.1099[M+Na]^+^  358.1041[M+H-CH_3_]^+^  343.0814[M+H-2CH_3_]^+^  325.0701[M+H-2CH_3_-H_2_O]^+^  315.0831[M+H-2CH_3_-CO]^+^  313.1029[M+H-4CH_3_]^+^ | nd | Sinensetin |
| 11 | 5.68 | C_21_H_22_O_8_ | 403.14066  (2.18) | nd | 425.1211[M+Na]^+^  388.1153[M+H-CH_3_]^+^  373.0923[M+H-2CH_3_] ^+^  358.0673[M+H-3CH_3_] ^+^  355.0817[M+H-2CH_3_-H_2_O] ^+^  345.0955[M+H-2CH_3_-CO] ^+^ | nd | Dihydroxy- tetramethoxyfavone Nobiletin |
| 12 | 5.84 | C_19_H_18_O_6_ | 343.11958  (1.84) | nd | 365.0992[M+Na]^+^  313.0708[M+H-2CH_3_]^+^  181.0166, 153.0184 | nd | 5,7,8,4’- Tetramethoxyfavone |
| 13 | 6.11 | C_22_H_24_O_9_ | 433.15152  (2.21) | nd | 455.1313[M+Na]^+^  403.1414[M+H-2CH_3_] ^+^  373.0929[M+H-4CH_3_] ^+^ | nd | 3,5,6,7,8,3‘,4‘-  Heptamethoxyfavone |
| 14 | 6.32 | C_20_H_20_O_8_ | 389.12433  (0.94) | nd | 411.1060[M+Na]^+^  374.0991[M+H-CH_3_] ^+`^  359.0767[M+H-2CH_3_] ^+^  341.0656[M+H-2CH_3_-H_2_O] ^+^ | nd | 5-Hydroxy-6,7,8,3’,4’- pentamethoxyfavone |
| 15 | 6.39 | C_20_H_20_O_7_ | 373.13061  (0.87) | nd | 395.1083 [M+Na]^+^  358.1051[M+H-CH_3_] ^+^  343.0817[M+H-2CH_3_] ^+^  328.0577[M+H-3CH_3_] ^+^  325.0710[M+H-2CH_3_-H_2_O] ^+^  315.0869[M+H-2CH_3_-CO] ^+^  313.1020[M+H-4CH_3_] ^+^ | nd | Tangeretin |
