# Supplementary material for: Functional Characterization of a Flavonoid Glycosyltransferase in Sweet Orange (Citrus sinensis)
Source: Front Plant Sci. 2018 Feb 15;9:166. doi: 10.3389/fpls.2018.00166 (PMC5818429; doi:10.3389/fpls.2018.00166)
Supplement: Supplementary file 4 [file Table_3.DOCX]

**Table S3.** MS Analyses of Products in Figure 6 and 8.

| **Peak no.** | **RT(min)** | **MS data on [M-H]^-^ (*m/z*)** | **MS^2^ focused on [M+H]^+^ (m/z)** | **identification** |
| --- | --- | --- | --- | --- |
| 1 | 2.83 | 433.176 | 433.176, 271.233 | Naringenin 7-*O*-glucoside |
| 2 | 2.78 | 465.140 | 465.140, 303.087 | Hesperetin 7-*O*-glucoside |
| 3 | 2.65 | 460.627 | 460.627, 299.051 | Diosmetin 7-*O*-glucoside |
| 4 | 2.96 | 463.311 | 463.311, 284.177, 255.397, 227.571, 151.124 | Quercetin 7-*O*- glucoside |
| 5 | 3.22 | 447.171 | 447.171, 284.399, 255.000, 227.777, 151.000 | Quercetin 3-*O*-glucoside |
| 6 | 2.62 | 609.000 | 609.000, 447.051, 285.291 | Kaempferol 3,7-*O*-diglucoside |
| 7 | 2.72 | 447.111 | 447.111, 287.253, 227.351, 151.113 | Kaempferol 3-*O*-glucoside, |
| 8 | 2.86 | 447.231 | 447.231, 285.300, 255.010, 227.113, 151.003 | Kaempferol 7-*O*-glucoside |
| 9 | 2.78 | 592.211 | 593.113, 441.13, 301.120 | quercetin 7-*O*-rhamnoside |
| 10 | 2.69 | 468.323 | 468.323, 303.000 | Kaempferol 7-*O*- rhamnoside |
| 11 | 3.45 | 593.110 | 593.110, 446.00, 301.00 | quercetin 7-*O*-rhamnoside |
| 12 | 3.65 | 623.000 | 623.000, 461.177, 298.139 | quercetin 7-*O*-glucoside |
| 13 | 3.81 | 463.231 | 463.001, 300.235, 179.326, 151.117 | Kaempferol 7-*O*-glucoside |
